# Reciprocal Innovation in Action: Adapting a Community-Based Care Model for Pregnant and Parenting Women in Kenya to Indiana

**DOI:** 10.5334/aogh.5052

**Published:** 2026-04-17

**Authors:** Michael Scanlon, Dominique Dumornay, Debra K. Litzelman, Justus E. Ikemeri, Anjellah Jumah, Leonce Jean-Baptiste, Jasmine Jackson, Marie Nicolle Joseph, Astrid Christoffersen-Deb, Julia Songok, Laura J. Ruhl

**Affiliations:** 1 DePauw University, Greencastle, Indiana, USA; 2 Indiana University Center for Global Health, Indianapolis, Indiana, USA; 3 Regenstrief Institute, Indianapolis, Indiana, USA; 4 Academic Model Providing Access to Healthcare (AMPATH), Eldoret, Kenya; 5 Haitian Association of Indiana, Indianapolis, Indiana, USA; 6 University of British Columbia, Vancouver, Canada; 7 Moi University, School of Medicine, Eldoret, Kenya

**Keywords:** reciprocal innovation, global learning, community health

## Abstract

*Background:* Reciprocal innovation and global learning emphasize mutual exchange, shared benefit, and co-creation between partners in high-, middle-, and low-income settings to address persistent global health inequities. Building on more than three decades of collaboration between Indiana University and the Academic Model Providing Access to Healthcare partnership in western Kenya, the IU Center for Global Health established a reciprocal innovation program to apply lessons from global partnerships to health challenges in Indiana.

*Objectives/Methods:* This article presents a case study of reciprocal innovation in action through the adaptation of Chamas, a community-based maternal and child health program developed in Kenya, for Haitian immigrant communities in Indianapolis.

*Findings:* We describe the co-design and early implementation of the Chamas-Indiana program using a five-stage process framework for reciprocal innovation: (1) building partnerships and trust with immigrant-serving organizations; (2) identifying shared challenges and priorities; (3) shared learning and adaptation of Chamas; (4) piloting and evaluation; and (5) reciprocal feedback with Kenyan partners.

*Conclusions:* By situating this work within a shared global health priority area of maternal and child health, the case illustrates how reciprocal innovation can strengthen health equity efforts, bridge global and local learning, and foster enduring partnerships rooted in trust, cultural responsiveness, and mutual learning.

## Introduction

Reciprocal innovation and global learning have emerged as important frameworks for advancing health equity in a globalized world [1, 2]. While global health has often been characterized by the flow of knowledge and resources from high‑income countries (HICs) to low‑ and middle‑income countries (LMICs), reciprocal innovation and global learning emphasize mutual exchange, shared benefit, and the recognition of expertise and innovations generated across diverse contexts [3]. These frameworks challenge hierarchical models of knowledge transfer by elevating innovations developed in LMICs that may hold relevance for addressing persistent health disparities across the globe [4–6].

Reciprocal innovation builds on related approaches such as “reverse innovation” and “frugal innovation,” which highlight the value of solutions developed in resource‑constrained environments for broader application [7]. Reciprocal innovation pushes these approaches forward by grounding this work in partnership and co‑creation. In other words, innovations are not simply transplanted from one context to another but are iteratively adapted through sustained collaboration, community engagement, and shared learning [8]. This emphasis on reciprocity reflects a growing recognition that health inequities are global in nature and require collective problem‑solving that transcends geographic and economic divides.

Indiana University Center for Global Health (IUCGH), alongside its partners at Moi University and the Academic Model Providing Access to Healthcare (AMPATH) in Kenya, developed the concept of reciprocal innovation based on our over 30‑year partnership in health education, research, and care [9]. Originally housed within Indiana’s Clinical and Translational Sciences Institute, IU, Purdue, and Notre Dame investigators established a reciprocal innovation program to leverage the power of AMPATH and similar global health partnerships to help address health challenges in Indiana. To co‑develop this program, partners from IUCGH and collaborating institutions in Indiana and Kenya engaged in a series of collaborative assessments and annual reflection meetings. Together, we launched a pilot grants program to support reciprocal innovation projects and appointed an inaugural Director of Reciprocal Innovation (LJR) to help steward this shared agenda. This institutional investment signals a shift toward integrating global learning into US health equity work and building durable infrastructures for bi‑directional exchange, which mirrors efforts at other institutions like University of Maryland Baltimore’s Global Learning for Health Equity Network.

**Figure 1:**
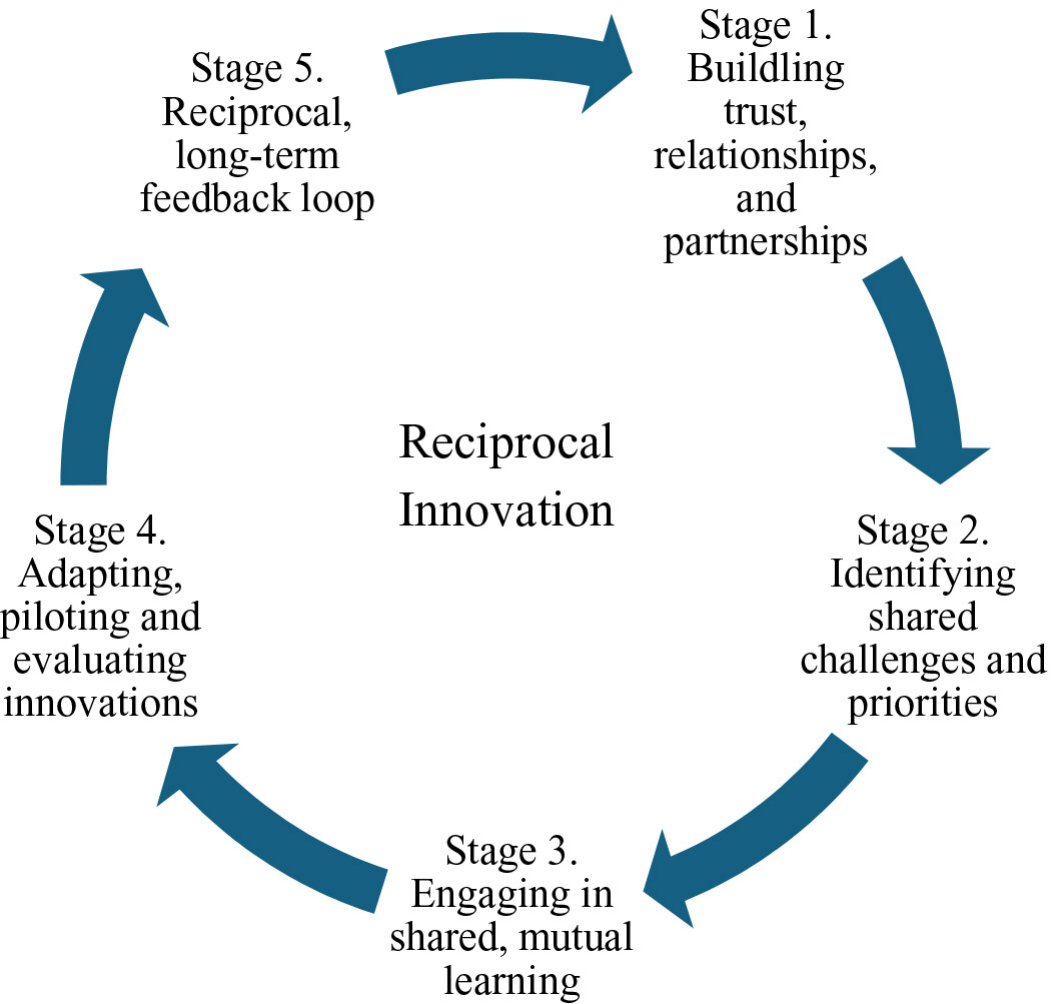
A process for reciprocal innovation of a community‑based maternal and child health program.

In this article, we describe a collaboratively developed case study that illustrates reciprocal innovation in practice. The process framework (Figure 1) traces the co‑learning journey undertaken by partners in Indianapolis and western Kenya through five interrelated stages: (1) cultivating relationships and trust with immigrant‑serving organizations and communities in Indianapolis; (2) identifying shared priorities and areas for collective action; (3) engaging in mutual learning around Chamas, a community‑based maternal and child health program originally co‑created in western Kenya; (4) jointly adapting, piloting, and evaluating the Chamas approach within immigrant communities in Indianapolis; and (5) sustaining a reciprocal exchange of insights with Indiana and Kenyan collaborators to inform ongoing refinement in both contexts. By focusing on maternal and child health, which was identified as a priority area of Haitian community partners in Indianapolis, the case study is situated within a global priority area with entrenched inequities in both LMIC and US settings and provides an opportunity to explore the potential and challenges of reciprocal innovation as a strategy for advancing global health equity.

## Reciprocal Innovation in Action: Adapting a Community‑Based Intervention from Kenya to Indiana

### Stage 1. Building partnership

A core philosophy of reciprocal innovation is building partnerships and trust with communities, yet building authentic relationships requires time, humility, and an alignment of shared interests and needs. To build our reciprocal innovation program, investigators at IUCGH who were engaged in work at AMPATH in Kenya explored community partnerships that might align with IUCGH’s areas of expertise locally in Indianapolis. Obviously, many existing community‑engaged programs and researchers were partnering with neighborhoods and organizations in Indianapolis. To avoid duplicating or competing with existing efforts, the IUCGH team looked for opportunities to partner with communities whose needs were significant but under‑engaged by academic and other partners. Immigrant communities emerged as a natural fit, given both the pressing health inequities they face, which are often exacerbated by discrimination, language barriers, and other forms of marginalization, and the expertise the IUCGH team, alongside Kenyan partners through AMPATH, brings in global health, cultural responsiveness, and cross‑cultural partnership.

One such community is the Haitian community in Indianapolis, which, like other Midwestern cities, has experienced a significant influx of Haitian people in the past several years. The Haitian Association of Indiana (HAI), a nonprofit community‑based organization established in 2008, has been working to promote the social well‑being of Haitians residing in Indiana and to recognize and celebrate Haitian culture through educational programming, cultural events, and community resource initiatives [10]. HAI serves as a vital liaison and hub for Haitian‑serving and immigrant‑serving organizations across Indiana. Over several months of meetings and sustained dialogue with HAI and its partners about shared values, priorities, and mutual commitment to collaboration, IUCGH decided to focus our initial reciprocal innovation activities on the Haitian community in Indiana. The collaboration with HAI opened doors to additional Haitian‑serving and immigrant‑serving organizations, laying the groundwork for a broader network of trust and engagement to support reciprocal innovation.

As a first step in building a shared program for reciprocal innovation, IUCGH and HAI co‑led the establishment of the Haitian Health Coalition, a coalition that brings together local community leaders and organizations, health and social service providers, and local and state health authorities to coordinate in addressing the needs of the growing Haitian community in Indianapolis. The Coalition met monthly during the first year of the partnership and allowed the coalition to identify and engage with different partners and communities who had the time and desire to collaborate in this work. In addition, IUCGH and HAI decided on a formal partnership model and memorandum of understanding to guide shared work. This included IUCGH renting space within the HAI offices to ensure that the program remained embedded within a community‑facing setting. Additionally, IUCGH supported HAI’s experienced community liaison to serve as a primary facilitator of our community engagement efforts. The liaison is of Haitian descent and is fluent in Haitian Creole. She has worked with various organizations in the United States and Canada to support the Haitian diaspora and has also collaborated with organizations within Haiti for many years.

### Stage 2. Identifying shared challenges and priorities

As the relationship between IUCGH, HAI, and other organizations and individuals developed, we began the process of identifying specific priority areas that the coalition could address. In consultations with community partners, persistent challenges were identified within the Haitian community in Indianapolis, including precarious immigration status, limited access to stable employment, inadequate housing, and difficulties navigating the healthcare system. In response, the coalition fostered collaboration and facilitated the exchange of ideas and best practices aimed at addressing these multifaceted issues. Insights gathered through focus groups and coalition meetings underscored a strong community interest in enhancing social cohesion and support structures, particularly during critical life stages such as pregnancy and early childhood. From discussions with Haitian community leaders and key health experts and partners, the coalition identified mental health and maternal and child health as two pressing health areas of concern.

The emergence of maternal child health as a priority for Haitian partners aligned well with IUCGH’s global expertise, given work across various maternal and child health programs at AMPATH in Kenya. Many of these programs have leveraged the power of community health workers to engage pregnant and parenting women in care, with the Chamas model representing a particular innovative and successful model in Kenya [11]. Maternal and child health has long been a central focus of global health policy and programming, reflecting its importance for population well‑being and development [12]. Despite substantial progress over recent decades, maternal mortality remains high in countries like Kenya. Neonatal and child health indicators similarly reflect persistent inequities, often exacerbated by poverty, health system constraints, and social determinants of health.

At the same time, the United States faces its own maternal health crisis, with maternal mortality rates that significantly exceed those of most other HICs. Racial inequities are stark, with Black women in the United States many more times likely to die from pregnancy‑related causes than White women [13]. Immigrant women, particularly those with limited English proficiency or uncertain immigration status, face additional barriers to care [14]. These inequities are compounded by fragmented health systems, limited access to culturally responsive services, and structural discrimination. While group care models led by a physician or nurse like CenteringPregnancy have been shown to be effective in the United States [15], most of these programs are clinic‑based and may not be accessible or acceptable to certain communities for a variety of reasons, including difficulty traveling to a clinic, language or cultural barriers, and inaccessible health systems [16]. Models that leverage community health workers in community‑based settings to support pregnant and parenting women are less common in the United States but have shown significant progress in global settings [17–19].

### Stage 3. Shared learning about and adaptation of a “chamas” intervention

#### Chamas program in kenya

Chamas was developed within the AMPATH program in western Kenya as an integrated group care and microfinance model for pregnant and parenting women. The intervention is rooted in the Swahili term *chamas*, which can be translated to mean “groups with a purpose,” reflecting the emphasis on collective learning, peer support, and social solidarity. Women are organized into small groups that meet regularly during their pregnancy and postpartum period, facilitated by trained community health workers. Sessions combine health education, peer support, and microfinance activities, creating a holistic platform for improving maternal and child health outcomes [11].

Evidence from randomized controlled trials and implementation studies has demonstrated the effectiveness of Chamas. Women who participated in Chamas were more likely to deliver in health facilities, complete recommended antenatal care visits, and practice exclusive breastfeeding compared to nonparticipants [20]. Beyond health outcomes, participants reported increased social support, empowerment, and financial resilience, highlighting the program’s broader impact on determinants of health. The integration of microfinance not only addressed economic barriers but also created mechanisms for sustainability, as women’s contributions to group savings reinforced ongoing participation and engagement.

Over the past decade, Chamas has been scaled across multiple regions in western Kenya and has been incorporated into government health strategies. The model has been recognized for its adaptability, low‑cost structure, and potential for integration into health systems. Its success reflects the co‑creation process of AMPATH, which has always emphasized partnership between Kenyan and US institutions, and between health systems and communities [9]. This combination of evidence, scalability, and community ownership positions Chamas as a promising candidate for reciprocal innovation.

#### Adaptation of the chamas intervention to indiana

We explored the potential for Chamas as an intervention model to address health and social needs among the Haitian community members in Indianapolis using several strategies. To learn about the Chamas program and its potential for adaptation in Indiana, IUCGH’s community liaison (DD) traveled to Kenya to spend a week with the Chamas team to learn directly from Kenyan facilitators, observe Chamas sessions, share about Haitian’s health journey in Indiana, and discuss lessons for adaptation. This exchange embodied the principle of reciprocal innovation, as Indiana partners were not simply importing an intervention but actively learning and adapting alongside Kenyan counterparts. For example, during a comprehensive full‑day session, the IUCGH‑AMPATH team identified that both pregnant women in western Kenya and Haitian‑born pregnant women in Indiana were particularly vulnerable, given complex social determinants and access to and navigation of healthcare systems. One shared critical barrier faced by these groups is limited access to prenatal care during the early stages of pregnancy. For reciprocal innovation, even given the common challenges and needs, it is imperative to adapt programs to align with the structure and nuances of local healthcare systems, while also ensuring cultural responsiveness for the Haitian community. The Kenyan team’s implementation of culturally attuned and effective interventions served as a valuable model.

The objective was to uphold similar standards and fidelity to the Chamas model while tailoring and adapting approaches to meet the specific needs of Haitian immigrant women in Indiana. Kenyan partners shared not only successes but also challenges in implementation, including issues of sustainability, participant retention, and balancing health and economic priorities. These insights were invaluable in shaping realistic expectations for adaptation in Indiana. The process also created opportunities for Kenyan partners to reflect on their own work, as questions from Indiana collaborators prompted new discussions about curriculum design, monitoring strategies, and potential areas for innovation.

To ensure relevance in Indiana and support adaptation of the program, we conducted focus group discussions with pregnant and parenting Haitian women in Indianapolis to understand their pregnancy and parenting experiences, health and social needs, and perspectives about a Chamas model for group‑based care. We conducted two focus group discussions with 20 Haitian pregnant and parenting women and another focus group discussion with a cohort of 10 community health workers who worked with pregnant and parenting women in Indianapolis as part of a program called WeCare [18]. Overall, participants were positive about a Chamas group care program in Indianapolis and valued the opportunity for peer support and relationship building with other women, as pregnancy was experienced by many as a socially isolating experience. Participants also felt like peer and group models could engage women who may otherwise be reluctant to engage or be linked to services, as they could have peers vouching for the program and activities. Participants emphasized the importance of wrap‑around services that addressed both health and social needs, such as English as a Second Language classes, job training, and assistance with navigating complex health and social service systems. These priorities informed modifications to the Chamas curriculum, including the integration of modules on health systems navigation and connections to social services. The adaptation process thus combined fidelity to the core elements of Chamas with flexibility to incorporate community‑identified priorities, ensuring both cultural relevance and program feasibility.

A community health worker of Haitian descent was recruited and hired full time to lead the pilot program and ensure culturally and linguistically appropriate services. Fluent in French, Haitian Creole, and Spanish, this individual brought necessary linguistic and cultural competencies to the program. Training for this position leveraged training resources from three different programs (an Indiana‑certified community health worker training program, the WeCare program that provides care for pregnant and parenting women in Indianapolis, and the Chamas Kenya program training), with specific content related to and resources to support pregnant and postpartum women, infants, and families at elevated risk for adverse birth outcomes, including low birthweight and infant mortality. A central component of this training was personalized coaching, which has been associated with measurable improvements across key maternal and infant health indicators, including reductions in low birthweight incidence and infant mortality rates [18].

### Stage 4. Piloting and evaluating an adapted chamas intervention in indianapolis

The IU‑HAI team is preparing to launch a pilot of Chamas in Indianapolis, with an initial cohort of pregnant women from the Haitian community. The pilot will evaluate feasibility and acceptability, using both qualitative and quantitative measures to assess participant engagement, satisfaction, and early health outcomes. Process evaluation will capture implementation challenges and adaptations in real time, allowing for iterative refinement. Our evaluation framework draws from implementation science, emphasizing both effectiveness and contextual fit. Metrics will include maternal health service utilization (e.g., prenatal visits, facility delivery, length of stay, and readmission), and infant health indicators (breastfeeding, birth weight, gestational age, and immunizations), and social determinants of health, including language acquisition and employment support. Findings from the pilot will inform decisions about potential scale‑up and will be shared with both Kenyan and Indiana partners to advance shared learning. As the Chamas adaptation progresses, ongoing evaluation and reflection will generate lessons for both Indiana and Kenya, reinforcing the principle that global health is most impactful when it is reciprocal.

### Stage 5. Reciprocal feedback loop with partners in kenya

A defining feature of reciprocal innovation is the establishment of sustained, bidirectional feedback loops that promote mutual learning and benefits. The Chamas adaptation in Indianapolis is being designed from the outset not as a one‑time transfer of knowledge but as the potential start of an ongoing and long‑term collaboration between the Kenyan Chamas team and new partners in Indianapolis, like HAI and other community‑based organizations in Indianapolis. Continuous engagement of the Kenyan team ensures that the pilot in Indiana remains grounded in the model’s core values of community ownership, women’s empowerment, and group‑based support, while adapting flexibly to a new cultural and health system context. Equally important, this exchange allows the Kenyan team to gain new insights from the Indiana adaptation process that can inform the next generation of Chamas programming in Kenya and beyond.

Throughout the implementation and evaluation phases, members of the Chamas Kenya leadership team will be actively engaged as technical advisors, thought partners, and co‑interpreters of emerging data. They have co‑created the development of fidelity and adaptation metrics, reviewed training materials for community health workers, and advised on challenges such as participant recruitment, retention, and balancing health and social components of the curriculum. Regular joint meetings between the IU and AMPATH Chamas teams in Kenya, held virtually and periodically in person, will facilitate shared review of preliminary findings, troubleshooting of implementation barriers, and exchange of strategies for sustaining group cohesion. This iterative engagement creates an active learning environment in which both teams refine their models in real time.

Reciprocal learning also flows in the opposite direction. The Kenyan Chamas team can apply lessons from the Indiana pilot to strengthen and diversify implementation strategies in Kenya. To sustain and strengthen this loop, several strategies are being pursued. First, the IU and AMPATH teams plan to establish a joint learning collaborative that meets quarterly to review pilot findings and discuss programmatic challenges. Second, reciprocal visits bringing Kenyan implementers to Indiana during the pilot and facilitating future visits by Indiana partners to Kenya will support shared understanding and cross‑training. Third, a shared repository for tools and training materials, as well as lessons learned, will be developed to promote transparency, collective ownership, and scalability across both settings. Finally, both teams will collaborate on joint manuscripts and presentations, ensuring that the knowledge generated through this process contributes to global scholarship on community‑based maternal health and reciprocal innovation.

Through this feedback loop, the Chamas Kenya and Indiana teams continue to model the principles of reciprocity, humility, and co‑creation that underlie this approach. Rather than a one‑directional transfer of innovation, the ongoing partnership represents a dynamic system of shared learning where each setting contributes to and benefits from the other’s experience. In doing so, this collaboration demonstrates how reciprocal innovation can serve as both a process and a philosophy for advancing global health equity through sustained, trust‑based partnership.

## Reflections and Next Steps

This case study demonstrates the potential for reciprocal innovation to serve as a pathway for addressing health inequities across global contexts. By adapting an evidence‑based intervention from Kenya to Indiana with continuous feedback and engagement across settings, we situate reciprocity, partnership, and adaptation as important reframings of global health as a shared endeavor. The process also underscores how US investments and engagements globally can offer critical insights for addressing local health disparities by generating new knowledge and innovations through global health collaborations. The process illustrated in Figure 1 shows a process that is far more linear and stepwise than much of our experience through this process, but overall, it represents well how the partnerships and activities moved through various stages. stage 1 that focuses on building trust and relationships is a long‑term one and one that was supported and deepened through each subsequent stage of the process. While this focus on this case study was on a single innovation (Chamas), the reciprocal innovation process is meant to show how an initial partnership developed around the adaptation and testing of one innovation can lead to additional trust and partnership that can then lead to expanded focus and activities. This is shown by the Stage 5 arrow representing a reciprocal, long‑term feedback loop within the Chamas intervention to connect back to Stage 1, representing trust, relationships, and partnership, which can then start again on identifying a new shared health challenge, and so on.

This case study also revealed important challenges and barriers to this work. Securing sustainable funding remains a challenge and particularly now in a policy environment where global health funding is under threat. While the central value underpinning our work is driven by addressing health inequities wherever they occur globally, we also understand that US investments in this work often rely on return on investment and benefit to US national and health security aims. We believe that reciprocal innovation can be an important lens through which to achieve both of these aims: a commitment to health equity while recognizing the tangible benefits that global health collaborations and reciprocity have for the health of Americans. Another challenge facing this type of work is the funding and institutional silos that often exist between global health and US‑focused health equity initiatives. This creates barriers to reciprocal innovation, requiring new models of training and collaboration that transcend disciplinary boundaries. Finally, ensuring robust and equitable engagement of Kenyan and other global partners during implementation in Indiana raises important questions about recognition, authorship, and benefit sharing across partners. While new communication technologies provide opportunities for real‑time engagement and feedback on adaptation and evaluation processes that can inform the Chamas program in Indiana and Kenya, we acknowledge that existing programs and funding often create fewer opportunities for Kenyan engagement in Indiana‑based projects compared to US investigator engagement in Kenya‑based projects.

## Conclusion

Looking ahead, reciprocal innovation offers a promising framework for reframing global health in the 21st century. By moving beyond unidirectional models of knowledge transfer, it affirms the value of community‑driven solutions, mutual learning, and shared responsibility for addressing global challenges and operationalizes global learning to advance health equity through authentic partnership, shared expertise, and co‑adaptation across contexts. By adapting Chamas, an evidence‑based Kenyan innovation, to meet the needs of Haitian immigrant women in Indiana, this collaboration shows how global health partnerships can evolve into vehicles for mutual learning and local impact. The process of partnership building, shared design, and iterative evaluation reflects a deliberate commitment to reciprocity: valuing community expertise, redistributing knowledge flows, and embedding equity into both process and outcome. As reciprocal innovation moves forward, sustaining engagement with Kenyan partners in interpretation, reflection, and bidirectional learning will remain essential to ensure ongoing co‑creation rather than transfer. Future efforts must also address structural barriers such as fragmented funding, disciplinary silos between “global” and “domestic” health, and the undervaluing of community‑engaged work. Ultimately, this case underscores that global health equity depends not on the direction of innovation flow, but on the depth of partnership and the integrity of shared learning. Reciprocal innovation provides a replicable pathway for transforming how universities and health systems collaborate to confront shared global health challenges.
